# Impact of coronal and sagittal hindfoot alignment on metatarsus primus elevatus in patients with rheumatoid foot deformities

**DOI:** 10.1186/s12891-025-09112-x

**Published:** 2025-10-06

**Authors:** Shinichi Mizuki, Keiko Tanaka, Yoshihiro Miyake, Yusuke Horita, Kensuke Oryoji

**Affiliations:** 1 The Center for Rheumatic Diseases, Japanese Red Cross Matsuyama Hospital, 1 Bunkyo, Matsuyama, Ehime, Matsuyama, 790-8524 Japan; 2 Department of Epidemiology and Public Health, Ehime University Graduate School of Medicine, Toon, Japan

**Keywords:** Metatarsus primus elevatus, Rheumatoid foot deformity, Hindfoot alignment

## Abstract

**Background:**

Typical foot deformity patterns of patients with rheumatoid arthritis (RA) include hallux valgus, claw toes, splay foot, flat foot, and hindfoot valgus deformities. However, some patients present deformities that are different from a typical pattern, such as metatarsus primus elevatus, described as dorsal elevation of the first metatarsal in relation to the lesser metatarsals. We speculated that metatarsus primus elevatus might be associated with calcaneal inclination and hindfoot varus alignment; however, studies on the association of hindfoot alignment with metatarsus primus elevatus in patients with RA are limited.

**Objective:**

To elucidate the impact of hindfoot coronal and sagittal alignment on metatarsus primus elevatus in patients with RA.

**Methods:**

We performed a retrospective analysis of weight-bearing anteroposterior and lateral radiographs of 58 patients (112 feet) with rheumatoid foot deformities who underwent surgery. The degree of metatarsus primus elevatus (dMPE) was assessed based on the distance between the dorsal cortical bones of the first and second metatarsals, as measured on lateral radiographs. The intermetatarsal angle between the first and second metatarsals (M1M2), calcaneal pitch, and the naviculocuboid (N/C) overlap ratio were assessed. Patients were divided into four subgroups representing dMPE quartiles (Q1–Q4) as closely as possible. Analysis of covariance was used to calculate the adjusted means of the radiographic parameters. Logistic regression was used to assess the association between clinical and radiographic parameters and the risk of being in the highest dMPE quartile (i.e., Q4).

**Results:**

The median dMPE in patients with RA was 2.0 mm (interquartile range, 0.2–5.4 mm). Analysis after adjusting for sex, age, body mass index, and disease duration revealed that the M1M2 angle and N/C overlap ratio in the Q4 subgroup were significantly smaller than those in the Q1 subgroup (*p* < 0.01 for both parameters). Only the N/C overlap ratio showed a significant inverse association with the risk of being in Q4 (adjusted odds ratio: 0.94, 95% confidence interval: 0.91–0.97).

**Conclusion:**

A subset of patients with RA exhibits metatarsus primus elevatus, which is associated with hindfoot alignment. Recognizing this less common deformity pattern is important when planning treatment strategies for the rheumatoid foot.

## Introduction

Rheumatoid arthritis (RA) is a systemic autoimmune inflammatory disease that involves not only joints but also systemic organs. The foot and ankle region is commonly involved in the early stages of RA, with a prevalence of 19–43% [1, 2]. In a cohort study of patients with RA, 53% complained of foot or ankle symptoms; [2] indeed, more than 90% of patients with RA have foot and ankle symptoms during their disease course [3]. Moreover, patients present a wide variety of rheumatoid foot deformity patterns [3–5]. Typical deformity patterns of the rheumatoid foot are hallux valgus, claw toes, splayed foot, flatfoot, and hindfoot valgus deformities. However, as we reported previously [6], some patients present atypical deformity patterns, such as metatarsus primus elevatus.

Metatarsus primus elevatus, which was first reported by Lambrinudi in 1938, involves dorsal elevation of the first metatarsal relative to the lesser metatarsals [7]. Subsequently, metatarsus primus elevatus, and its specific role in the pathogenesis of hallux rigidus has remained an issue of debate [8–10]. Vainio reported that hallux rigidus in patients with RA is largely secondary to metatarsus primus elevatus. He also mentioned that metatarsus primus elevatus results from forefoot supination and a midfoot dorsiflexion deformity. However, he did not present any radiographic measurements to support this [3]. 

The patient with RA in our previous case report had hallux valgus and clawing of the lesser toes, but splayed foot was not observed [6]. Radiographic imaging revealed that the patient had a large calcaneal pitch (20°) and a small naviculocuboid overlap ratio (27%). These parameters indicate that the patient did not have flatfoot or valgus hindfoot alignment, which are typical rheumatoid deformities. These findings led us to speculate that metatarsus primus elevatus is associated with a large calcaneal pitch and varus hindfoot alignment. However, no reports about the aetiology of metatarsus primus elevatus in patients with RA have included radiographic assessment.

Here, we hypothesise that coronal and sagittal hindfoot alignment are associated with metatarsus primus elevatus. To assess this hypothesis, we analysed radiographic measurement parameters in patients with rheumatoid foot deformities who underwent surgical treatment.

## Methods

### Study design and patients

In this retrospective cohort study, patients with RA who underwent surgery for rheumatoid foot deformities, which caused gait disturbances, painful callosities on the feet, or both, were enrolled. Preoperative radiographs were analysed. Feet with a history of previous surgery or fracture, or feet with a neuromuscular disorder were excluded. We included both operative and nonoperative feet of patients. Specifically, 24 feet on the nonoperative side were included from patients who underwent surgery on the contralateral side. Healthy controls comprised patients who were referred to our department but not diagnosed with an inflammatory rheumatic disease. Healthy control participants with a history of previous foot surgery or fracture, apparent foot deformity (e.g., hallux valgus), or neurologic disorder were excluded. This study was conducted in accordance with the Declaration of Helsinki and approved by the Institutional Review Board of Japanese Red Cross Matsuyama Hospital (identification number: 844).

### Radiographic evaluations

Anteroposterior and lateral weight-bearing foot radiographs were obtained using a standardised technique. All radiographic parameters were measured by a single investigator (S.M.) to eliminate interobserver error (intraclass correlation coefficient: 0.85–0.97); the investigator was blinded to the clinical data, including disease activity and the RA treatment regimen.

**Fig. 1:**
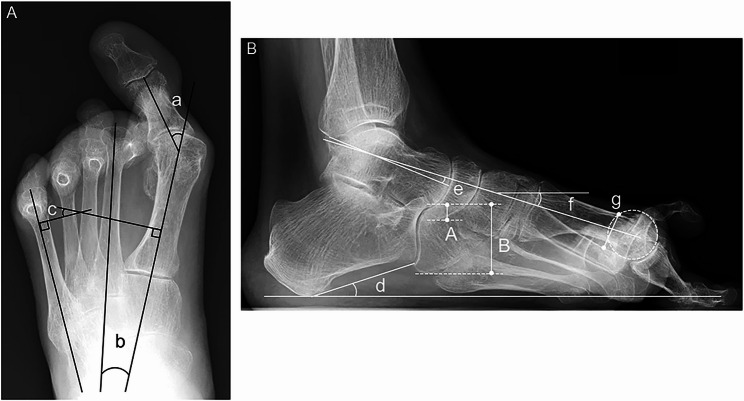
**A** a, hallux valgus (HV) angle; b, 1–2 intermetatarsal (M1M2) angle; c, 1–5 intermetatarsal (M1M5) angle. **B** d, calcaneal pitch; e, talar–first metatarsal (MT-1) angle; f, MT-1 declination angle; g, the degree of metatarsus primus elevatus (dMPE) is defined as the elevation of the first versus second metatarsal; A/B, naviculocuboid (N/C) overlap ratio

Three angles were measured on anteroposterior foot radiographs (Fig. 1A): the hallux valgus (HV) angle, the 1–2 intermetatarsal (M1M2) angle, and the 1–5 intermetatarsal (M1M5) angle. The HV angle was defined as the angle between the longitudinal axis of the first metatarsal shaft and the longitudinal axis of the proximal phalanx [11]. The M1M2 angle was defined as the angle between the longitudinal axis of the first and second metatarsal shafts [11], and the M1M5 angle was defined as the angle between the longitudinal axis of the first and fifth metatarsal shafts [12].

From the lateral foot radiographs, the following parameters were measured (Fig. 1B): degree of metatarsus primus elevatus (dMPE), calcaneal pitch, the first metatarsal (MT-1) declination angle, the talar–MT-1 angle, and the naviculocuboid (N/C) overlap ratio. To assess metatarsus primus elevatus, a circle was fitted over the first metatarsal head, ensuring congruency with the joint surface. The distance from the proximal intersection of the circle and the dorsal cortical bone of the first metatarsal to the dorsal cortical bone of the second metatarsal was measured to obtain the dMPE [10]. The calcaneal pitch was defined as the angle between the line drawn from the plantar surface of the calcaneus tuberosity to the plantar prominence of the calcaneus proximal to the calcaneocuboid joint and a line parallel to the floor [13]. The MT-1 declination angle was defined as the angle between the longitudinal axis of the first metatarsal bone, identified by connecting the midpoints of the distal and proximal dorsal and plantar margins of the diaphysis of the first metatarsal and a line parallel to the floor [8]. The talar–MT-1 angle was defined as the angle between the long axis of the talus and the long axis of the first metatarsal [14]. The N/C overlap ratio was defined as the height of the overlapping portions of the navicular and cuboid divided by the vertical height of the cuboid [15]. 

### Statistical analysis

Patients were divided into four subgroups based on dMPE values to represent quartiles as closely as possible: Q1, dMPE < 0.3 mm (*n* = 28); Q2, dMPE from 0.3 to < 2.0 mm (*n* = 26); Q3, dMPE from 2.0 to < 5.4 mm (*n* = 30); and Q4, dMPE > 5.4 mm (*n* = 28). Analysis of covariance was used to calculate the mean values of the radiographic parameters, adjusted for the following confounding factors: age, sex, body mass index, and RA disease duration. Logistic regression analysis was performed to assess the association between clinical and radiographic parameters and the risk of being in the highest dMPE quartile (i.e., Q4). A p value of < 0.05 was considered significant. All statistical analyses were performed using the SAS software package version 9.4 (SAS Institute, Cary, NC, USA).

## Results

### Characteristics of the study participants

Demographic characteristics of the patients are summarised in Table 1. A total of four out of 116 feet in 58 patients with RA were excluded in accordance with the exclusion criteria. Next, we compared 112 feet in patients with RA with 22 feet in the healthy control group.

**Table 1:** Demographic characteristics of patients with rheumatoid arthritis and healthy control participants

Variable	RA	Healthy controls
	112 feet; 58 patients	22 feet; 11 participants
Age (years)	67.1 (7.4)	51.6 (7.3)
Female, n (%)	103 (91.7)	20 (90.9)
Disease duration (years)	21.7 (9.1)	NA
Body height (cm)	149.6 (7.7)	158.7 (9.6)
Body weight (kg)	46.8 (8.1)	61.8 (16.5)
BMI (kg/m^2^)	20.9 (3.4)	24.4 (5.4)
Stage^*^ III/IV, n (%)	67 (59.8)/45 (40.2)	NA
RF positive (%)	95.8	NA
ACPA positive (%)	89.2	NA
MTX therapy, n (%)	86 (76.9)	NA
MTX weekly dose (mg)	7.2 (1.7)	NA
PSL therapy, n (%)	88 (78.6)	NA
PSL dose (mg/day)	4.3 (2.2)	NA
Biologic therapy, n (%)	31 (27.7)	NA
SDAI†	5.8 (3.4, 9.6)	NA
mHAQ†	0.25 (0.125, 1.00)	NA

Values are expressed as the means (SDs) unless otherwise indicated

*ACPA* anti-citrullinated peptide antibody, *BMI* body mass index, *mHAQ* modified Health Assessment Questionnaire, *MTX* methotrexate, *NA* not applicable, *PSL* prednisolone, *RA* rheumatoid arthritis, *RF* rheumatoid factor, *SDAI* simple disease activity index

† Values are expressed as the medians (interquartile ranges)

* Steinbrocker radiographic stage

### Distribution of dMPE values

**Fig. 2:**
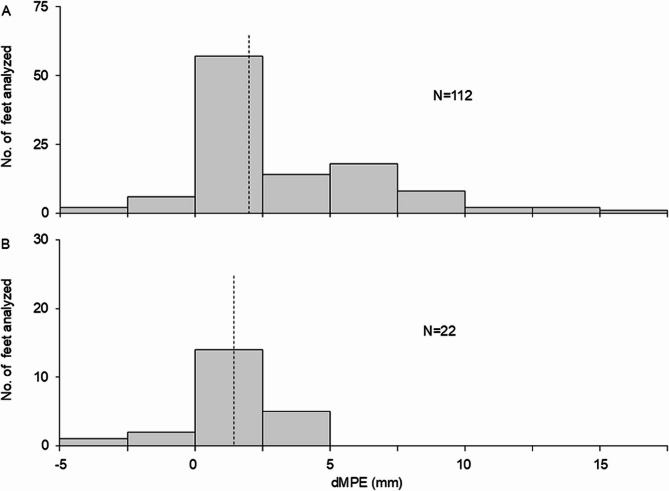
Distribution of dMPE values in (**A**) patients with rheumatoid arthritis and (**B**) healthy control participants. Vertical dashed lines indicate median values. dMPE, degree of metatarsus primus elevatus

In patients with RA who underwent surgical treatment for rheumatoid foot deformities, the median dMPE was 2.0 mm (interquartile range, 0.3–5.4 mm) (Fig. 2A). In the healthy control group, the median dMPE was 1.4 mm (interquartile range, 0–2.4 mm) (Fig. 2B).

### Feet of patients with RA compared according to dMPE quartile

We examined differences among the four subgroups of patients with RA according to dMPE quartile (Table 2). The M1M2 angle and N/C overlap ratio were significantly smaller (*p* < 0.01 for both parameters), and the calcaneal pitch was significantly larger (*p* = 0.02) in the Q4 subgroup than in the Q1 subgroup. However, no significant difference was observed between the Q1 and Q2 subgroups or the Q1 and Q3 subgroups. After adjusting for age, sex, body mass index, and RA disease duration, significant differences in the M1M2 angle and N/C overlap ratio between the Q1 and Q4 subgroups remained (*p* < 0.01 for both parameters); however, the difference in the calcaneal pitch was no longer significant.

**Table 2:** Comparison of radiographic parameters among patients with rheumatoid arthritis according to dMPE quartile subgroup

		Crude			Adjusted*		
	Quartile	Mean	SE	p	Mean	SE	p
HV angle	1	43.1	3.1	Reference	42.7	3.2	Reference
	2	42.2	3.3	1.00	42.8	3.3	1.00
	3	45.5	3.0	0.90	46.0	3.0	0.80
	4	35.1	3.1	0.18	34.4	3.2	0.18
M1M2 angle	1	14.4	1.0	Reference	14.6	0.9	Reference
	2	14.5	0.9	1.00	14.7	0.8	1.00
	3	12.8	0.8	0.40	12.8	0.7	0.27
	4	10.3	0.8	< 0.01	9.9	0.8	< 0.01
M1M5 angle	1	35.0	1.5	Reference	35.6	1.5	Reference
	2	36.3	1.4	0.85	36.4	1.4	0.97
	3	34.3	1.2	0.97	34.4	1.2	0.85
	4	33.3	1.3	0.73	32.6	1.4	0.33
Calcaneal pitch	1	14.8	1.1	Reference	15.0	1.1	Reference
	2	13.6	1.1	0.77	13.8	1.1	0.80
	3	15.6	1.0	0.91	15.7	1.0	0.94
	4	18.9	1.1	0.02	18.4	1.1	0.07
N/C overlap ratio	1	63.6	3.4	Reference	63.5	3.5	Reference
	2	55.0	3.5	0.19	55.7	3.5	0.28
	3	52.9	3.2	0.06	52.9	3.2	0.07
	4	39.1	3.5	< 0.01	38.6	3.7	< 0.01
MT-1 declination angle	1	23.4	0.8	Reference	23.6	0.8	Reference
	2	22.9	0.8	0.97	22.9	0.8	0.89
	3	24.0	0.8	0.91	24.0	0.8	0.97
	4	23.2	0.8	1.00	22.9	0.8	0.87
Talar–MT-1 angle	1	−8.2	1.7	Reference	−8.0	1.8	Reference
	2	−7.8	1.8	1.00	−7.5	1.8	1.00
	3	−4.4	1.6	0.28	−4.4	1.7	0.32
	4	−3.3	1.8	0.13	−3.8	1.9	0.27

p values for comparison with the Q1 group were calculated using an analysis of covariance model

*dMPE* degree of metatarsus primus elevatus *HV angle* hallux valgus angle, *M1M2 angle* 1–2 intermetatarsal angle, *M1M5 angle* 1–5 intermetatarsal angle, *MT-1* first metatarsal, *N/C* naviculocuboid, *SE* standard error

*Adjusted for sex, age, body mass index, and disease duration

† p<0.05 vs. reference group (Q1)

†† p<0.01 vs. reference group (Q1)

### Risk for being in the highest dMPE (Q4) subgroup among patients with RA

Table 3 shows the crude and adjusted odds ratios and 95% confidence intervals (CIs) for being in the highest quartile of dMPE (Q4) according to clinical or radiographic parameters. A crude analysis revealed that the calcaneal pitch was associated with an increased risk of Q4 status. In contrast, the N/C overlap ratio and age were significantly associated with a decreased risk of Q4 status. After all of the variables listed in Table 3 were adjusted, only the N/C overlap ratio showed a significant inverse relationship with the risk of Q4 status (adjusted odds ratio, 0.94; 95% CI, 0.91–0.97).

**Table 3:** Logistic regression analysis for being in the highest dMPE quartile (Q4) among patients with rheumatoid arthritis

Parameter	Crude OR (95% CI)	Adjusted* OR (95% CI)
Calcaneal pitch	1.14 (1.05–1.25) †	1.10 (0.99–1.23)
N/C overlap ratio	0.95 (0.92–0.97) †	0.94 (0.91–0.97) †
Age	0.93 (0.87–0.99) †	0.93 (0.86–1.01)
Sex	0.38 (0.09–1.64)	0.21 (0.03–1.41)
BMI	1.03 (0.91–1.17)	0.99 (0.83–1.18)
Disease duration	1.00 (0.95–1.05)	1.02 (0.95–1.09)

*BMI* body mass index, *CI* confidence interval, *dMPE* degree of metatarsus primus elevatus, *N/C* naviculocuboid, *OR* odds ratio

* Adjusted for age, sex, BMI, and disease duration

### Comparison between the feet of healthy control participants and those of patients with RA

**Fig. 3:**
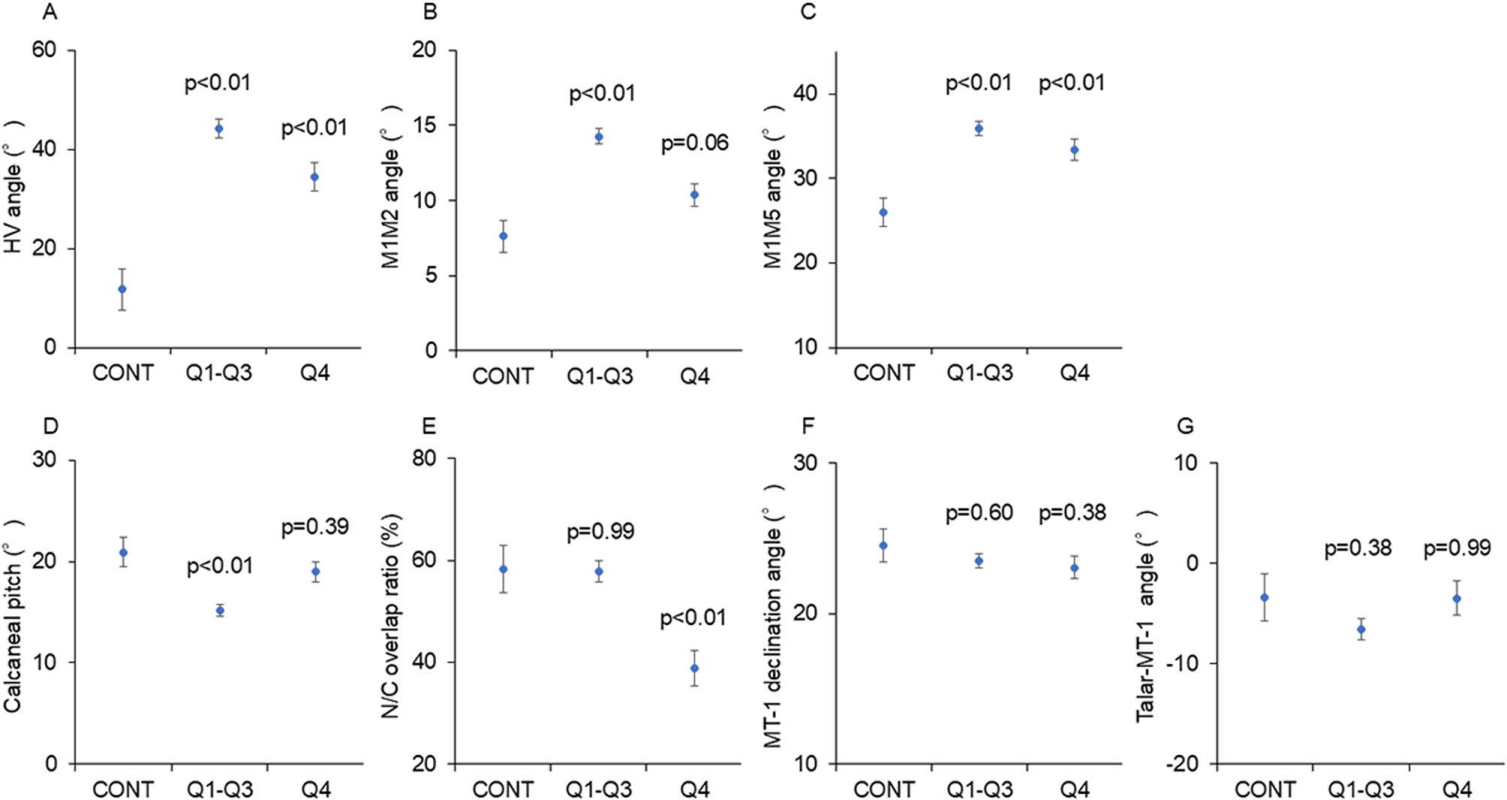
Comparison of radiographic parameters among the Q1–Q3, Q4, and healthy control groups (**A**) HV angle, (**B**) M1M2 angle, (**C**) M1M5 angle, (**D**) calcaneal pitch, (**E**) N/C overlap ratio, (**F**) MT-1 declination angle, and (**G**) talar–MT-1 angle. CONT, healthy control group; HV angle, hallux valgus angle; M1M2 angle, 1–2 intermetatarsal angle; M1M5 angle, 1–5 intermetatarsal angle; MT-1, first metatarsal; N/C, naviculocuboid; Q1–3, first, second, and third dMPE (degree of metatarsus primus elevatus) quartiles; Q4, highest dMPE quartile. P values for comparison between the Q1–Q3 subgroups or Q4 subgroup and the healthy control group were calculated using an analysis of covariance model adjusted for sex, age, and body mass index

When all study participants were divided into three groups (healthy controls, Q1–Q3, and Q4), the Q1–Q3 and Q4 groups had significantly larger HV (*p* < 0.01 for both) and M1M5 angles (*p* < 0.01 for both) than the healthy control group (Fig. 3A, C). The M1M2 angle in the Q1–Q3 group was significantly greater than that in the healthy control group (*p* < 0.01), but no difference was observed between the Q4 group and the healthy control group (Fig. 3B). Compared with the healthy control group, the Q1–Q3 group had a significantly smaller calcaneal pitch (*p* < 0.01), which was not observed in the Q4 group (Fig. 3D). The N/C overlap ratio in the Q4 group was significantly lower than that in the healthy control group (*p* < 0.01) but was similar between the Q1–Q3 and the healthy control groups (Fig. 3E). No difference was observed in the MT1 declination angle or talar–MT-1 angle among the three groups (Fig. 3F, G).

## Discussion

### Prevalence of metatarsus primus elevatus

As shown in Fig. 2, the prevalence of metatarsus primus elevatus in patients with RA who underwent surgical treatment was low. Although there is no reliable threshold value for pathologic dMPE, two previous studies that used the same method of measurement as the present study reported mean dMPE values of 2.0 ± 1.8 mm [16] and 2.6 mm (95% CI, 2.1–3.2 mm) [10], respectively. If we use the threshold value reported by Bouichae et al. [10]., then only 30 feet (26.8%) in the present study had a dMPE > 5 mm, and no feet in the healthy control group had a dMPE > 5 mm. Only the study by Matsumoto et al. measured dMPE in patients with RA. They found that fewer than 5% of patients with RA had a dMPE > 8.4 mm [16]. The prevalence of dMPE > 8.4 mm in the present study (9.8%) is similar to that reported by Matsumoto et al. Thus, metatarsus primus elevatus is an atypical rheumatoid foot deformity.

### Radiographic parameter analysis

The radiographic parameters in the Q1, Q2, and Q3 subgroups were not significantly different (Table 2). The axial alignment parameters and calcaneal pitch in the Q1–Q3 subgroups were significantly different than those in the healthy control group (Fig. 3A–D), suggesting that the Q1–Q3 subgroups had typical rheumatoid foot deformities such as HV, splayed foot, and flatfoot, which are often encountered in daily clinical practice. The Q4 subgroup had a smaller N/C overlap ratio than the Q1–3 and healthy control groups. These results may indicate that, in the case of typical rheumatoid foot deformities, the hindfoot is extremely flattened across a wide range of coronal alignments, but metatarsus primus elevatus may be due to supination of the hindfoot with normal sagittal calcaneal alignment. No studies have investigated this association. Some studies that used different methods to investigate dMPE in patients with RA [16] or hallux rigidus [8, 10, 17] did not measure the N/C overlap ratio. The results of the present study support our hypothesis that hindfoot alignment affects metatarsus primus elevatus, a forefoot deformity in patients with RA. Therefore, further investigation using other methods of measurement, i.e., tibia-hindfoot angle in the hip-to-calcaneal view [18, 19] or 3-dimensional computed tomography [20], is required.

### Aetiology of metatarsus primus elevatus

Little is known about the aetiology of metatarsus primus elevatus, which has been discussed vigorously in the literature regarding hallux rigidus. Some authors describe metatarsus primus elevatus as being caused by avoidance of loading painful medial rays or lesser metatarsal heads [3, 21]. A decreased medial longitudinal arch could increase tension in the intrinsic foot muscles and the plantar aponeurosis, possibly contributing to flexion contracture at the first MTP joint, which might in turn influence hindfoot alignment. However, in our study, patients in the Q4 group did not show flexion contracture at the first MTP joint. Additionally, the calcaneal pitch angle in the Q4 group was significantly greater than that in the Q1–Q3 groups and similar to that in healthy controls, suggesting preserved medial arch height. These findings make the proposed mechanism less likely in our cohort.

We also acknowledge the theoretical possibility that hallux deformity could precede and affect hindfoot alignment. Previous reports support the idea that hindfoot deformity can precede and aggravate hallux valgus. Hirao et al. [22] described cases in which progression of hindfoot valgus deformity led to worsening of hallux valgus, and Yamada et al. [23] reported correction of hallux valgus following surgical correction of hindfoot valgus. These findings suggest a directional relationship from proximal (hindfoot) to distal (forefoot), and we consider that a similar pattern may be involved in the development of metatarsus primus elevatus. Furthermore, in the study by Mizuki et al., metatarsal primus elevatus was surgically corrected using a plantar flexion wedge osteotomy, but no changes in hindfoot alignment were observed postoperatively. This may indicate that forefoot surgery alone does not affect hindfoot alignment in this deformity pattern. The potential for hindfoot realignment to correct forefoot deformities warrants further investigation.

Moreover, increased tension in the plantar aponeurosis [24, 25] or intrinsic muscles of the great toe [21] and an imbalance between the extrinsic and intrinsic muscles [26] can cause metatarsus primus elevatus. Notably, although the Q4 subgroup showed significantly reduced N/C overlap ratios, indicating hindfoot varus on average, not all patients in this group presented varus alignment. According to the thresholds defined by Lee et al. [15], 4 out of 23 feet in the Q4 subgroup fell within the range of hindfoot valgus. This finding suggests that metatarsus primus elevatus may result from multiple aetiologies in RA patients. We carefully excluded patients with hallux rigidus, neuromuscular disorders, or congenital clubfoot through clinical interviews and physical examination. However, we acknowledge that radiographic analysis alone cannot fully capture soft tissue pathology. Further studies, including assessments of muscle function, tendon condition, and joint/tendon sheath inflammation, are warranted.

### Study limitations

This study has some limitations. First, this was a cross-sectional study. Several reports have asked whether hindfoot alignment affects the outcome of surgery for rheumatoid forefoot deformity [27–29]. Longitudinal changes in metatarsus primus elevatus and its association with hindfoot alignment need further investigation to elucidate the natural history or postoperative course. A second limitation was the 2-dimensional nature of the analysis. Points projected on film may represent anatomically distinct points depending on their three-dimensional positioning, for example, hindfoot valgus and varus; therefore, 3-dimensional computed tomography data might be more suitable for analysis. Third, this study included non-surgically treated feet. In a subgroup analysis limited to nonoperative feet, we found that the N/C overlap ratio in the Q4 group remained significantly reduced, consistent with our main findings. Finally, this study included only patients who had undergone foot surgery. Future studies should include patients at various stages of disease progression.

## Conclusions

Our study reveals that metatarsus primus elevatus may occur in a subset of patients with RA and is associated with hindfoot alignment. Recognizing this less common deformity pattern may contribute to more individualized and effective treatment strategies in complex rheumatoid foot pathology.

## Data Availability

The datasets used and/or analysed during the current study are available from the corresponding author upon reasonable request.

## Abbreviations

RA: Rheumatoid arthritis
HV: Hallux valgus
M1M2: 1–2 intermetatarsal
M1M5: 1–5 intermetatarsa
dMPE: Degree of metatarsus primus elevatus
MT-1: First metatarsal
N/C: Naviculocuboid
